# Biophysical effects, safety and efficacy of raspberry leaf use in pregnancy: a systematic integrative review

**DOI:** 10.1186/s12906-021-03230-4

**Published:** 2021-02-09

**Authors:** Rebekah Bowman, Jan Taylor, Sally Muggleton, Deborah Davis

**Affiliations:** 1 University of Canberra, 11 Kirinari St, Bruce ACT, 2617 Australia; 2 ACT Government, Health Directorate, 4 Bowes St, Phillip ACT, 2606 Australia

**Keywords:** Pregnancy, Herbal, Complementary medicine, midwifery, Evidence-based practice

## Abstract

**Background:**

Childbearing women have been using various herbs to assist with pregnancy, labour and birth for centuries. One of the most common is raspberry leaf. The evidence base for the use of raspberry leaf is however under-developed. It is incumbent on midwives and other maternity care providers to provide women with evidence-based information so they can make informed choices. The aim of this study was to review the research literature to identify the evidence base on the biophysical effects, safety and efficacy of raspberry leaf in pregnancy.

**Methods:**

A systematic, integrative review was undertaken. Six databases were searched to identify empirical research papers published in peer reviewed journals including in vitro, in vivo, human and animal studies. The search included the databases CINAHL, MEDLINE, Cochrane Library, Scopus and Web of Science Core Collection and AMED. Identified studies were appraised independently by two reviewers using the MMAT appraisal instrument. An integrative approach was taken to analysis.

**Results:**

Thirteen studies were included. Five were laboratory studies using animal and human tissue, two were experiments using animals, and six were human studies. Included studies were published between 1941 and 2016. Raspberry leaf has been shown to have biophysical effects on animal and human smooth muscle including the uterus. Toxity was demonstrated when high doses were administered intravenously or intaperitoneally in animal studies. Human studies have not shown any harm or benefit though one study demonstrated a clinically meaningful (though non-statistically significant) reduction in length of second stage and augmentation of labour in women taking raspberry leaf.

**Conclusions:**

Many women use raspberry leaf in pregnancy to facilitate labour and birth. The evidence base supporting the use of raspeberry leaf in pregnancy is weak and further research is needed to address the question of raspberry leaf’s effectiveness.

## Background

Midwives have been using herbs in their practice for centuries. Martha Ballard for example was an eighteenth-century midwife who attended almost one thousand births in her long career. Her diary references the use of many different herbs and illustrates the primary ritual of her practice involving the gathering of remedies from the earth [1]. While midwifery has evolved since the time of Martha Ballard, the use of herbs in pregnancy remains widespread, particularly parturients (such as raspberry leaf) which are herbs that are thought to aid childbirth [2]. It is commonplace for women to seek guidance from midwives regarding the use of herbs during pregnancy [3, 4] and it is incumbent on midwives and other maternity care providers to assist women to make well informed decisions. The critical issue for the contemporary midwife is the evidence base for the use of such preparations.

The use of herbs in pregnancy can be a part of Complementary and Integrative Medicine (CIM) which is defined by the National Centre for Complementary and Integrative Health as a health care approach outside of mainstream Western or conventional medicine [5]. Large surveys in Australia found between 52 and 73% of pregnant women were using CIM and 37 to 48% consulted a CIM practitioner (for example a naturopath or herbalist) through their pregnancy [6, 7]. Raspberry leaf (*Rubus idaeus* of the Rosacea family) was found to be one of the top five herbs being used by pregnant women and being prescribed by CIM practitioners. Forster, et al. [8] identified that 36% of women attending a public antenatal clinic in Melbourne Australia took at least one herbal supplement during pregnancy, with the most common being raspberry leaf (14%). Mollart, et al. [9] found that 52.5% of a group of Australian midwives (*n* = 571) recommended raspberry leaf to women experiencing a post-dates pregnancy and incidentally, it was also the most frequently used CIM strategy in their own pregnancies.

Raspberry leaf is frequently used during pregnancy and labour to strengthen and tone the uterus, theoretically assisting contractions and preventing haemorrhage [10, 11]. While there is a long history of raspberry leaf use in pregnancy there is little research contributing to the evidence base especially in relation to its mechanism of action [12, 13], efficacy or potential harmful effects [11]. One review of scientific literature exploring raspberry leaf use in pregnancy [14] (now more than 10 years old) concluded that there was not enough evidence to recommend its use in this context. Focusing more broadly on herbal remedies used by pregnant women, Dante, et al. [15] conducted a systematic review of epidemiological studies into herbal therapies that included two studies on raspberry leaf [16, 17]. The findings relating to raspberry leaf use in pregnancy were inconclusive.

The National Institute for Health Care and Excellence (NICE) guidelines [18] recommend further research to evaluate effectiveness, safety and maternal satisfaction of the use of herbal supplements. In the meantime, midwives and other maternity care providers must draw on the evidence available and it is here that this paper contributes. The systematic integrative review presented here includes a broad range of research designs, studies conducted in both animals and humans and includes studies conducted since the review published by Holst in 2009 [14]. This integrative review therefore presents the current state of the art in relation to the evidence base informing the use of raspberry leaf in pregnancy.

## Methods

The aim of this systematic integrative review was to examine the research literature to identify the evidence base on the biophysical effects, safety and efficacy of raspberry leaf in pregnancy. A systematic integrative approach was taken. An integrative review includes diverse data sources which enhance a holistic understanding of the topic of interest. This method allows for inclusion of diverse methodologies, including experimental and non-experimental, and presents varied perspectives on the topic under study [19].

### Search strategy

The search was conducted in January 2019 in five databases: CINAHL, MEDLINE, Cochrane Library, Scopus, Web of Science Core Collection. The search was replicated in these and the AMED database in June 2020. Each database was searched using the search terms “raspberry leaf” AND (pregnan* OR labor OR labour OR uterus OR uterine OR birth). Manual searching of citations of identified papers was also conducted. Two authors independently conducted the search and selection of studies based on the eligibility criteria and aim of the study. The full research team met regularly to review the process and resolve any queries.

### Eligibility criteria

Research articles in peer review journals were included if they were in English, contributed to the aim which focused on biophysical effects, safety and efficacy of raspberry leaf use. Observational and experimental studies were included. Animal studies were included where they contributed to an understand of the potential biophysical effects as were in vivo and in vitro studies. No date limits were applied. Studies focussed on maternity caregivers’ or childbearing women’s experiences of raspberry leaf were excluded as were prevalence studies, commentaries, opinion pieces and reviews.

### Quality appraisal

Quality appraisal was undertaken using the Mixed Methods Appraisal Tool (MMAT) – version 2018. The MMAT is designed to include qualitative, quantitative and mixed methods studies in a complex literature review [20]. This tool was only used for the human studies in the review, as it was not found to be appropriate for in vitro studies and in vivo animal studies. Tools for appraising research quality for in vivo and in vitro studies are still emerging [21] and no useful tool could be identified. Two reviewers independently assessed each article and met to discuss the appraisal and resolve differences. A third reviewer was available to assess any disagreements not resolved through discussion though this was not required. No articles were excluded based on quality appraisal though this information has contributed to the assessment of the evidence base overall informing raspberry leaf use in pregnancy.

### Data extraction and analysis

Salient features of included studies were extracted in tabular format facilitating analysis and comparison across studies. The data extraction table included information on study design, sample, methods, dose and form of raspberry leaf used where reported, analysis, findings, critique and comments. Table 2 summarises the characteristics of included studies and main findings. A constant comparison method was the overarching approach taken to data analysis [19]. Extracted data were grouped and categorised by design and sample; for example in vitro studies examining effects on raspberry leaf on animal or human tissue, in vivo studies in animals and humans. This process facilitated analysis and comparison of findings for similar sorts of studies.

**Fig. 1:**
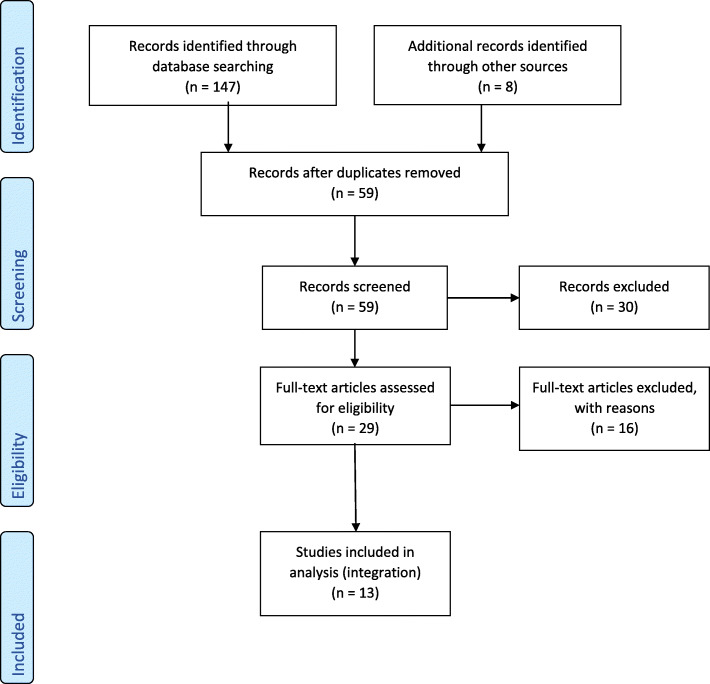
PRISMA flow diagram

## Results

### Search results

The initial database searches extracted 147 articles. An additional eight articles were identified from searching citations in identified articles. After removing duplicates, 59 articles remained. Thirty records were excluded prior to full text review; 19 as they did not report on safety or efficacy of raspberry leaf but rather prevalence of use, 10 as they were reporting on agriculture findings of the herb or other members of the Rubus family, and 1 as it was a theoretical discussion of the constituents and actions. Sixteen articles were excluded after full text review. These are presented with reasons for exclusion in Table 1. This left 13 articles to be integrated for this review. Figure 1 presents the PRISMA flow chart [47] illustrating the process.

**Table 1:** Excluded studies with reasons

Date, Author	Reason excluded
2000, Wilkinson [34]	A literature review on many herbs for morning sickness. No evidence presented on mechanism of raspberry leaf.
1999, McFarlin et al. [35]	Survey of herb used by midwives for induction. No evidence presented on mechanism of raspberry leaf.
2000, Parsons et al. [26]	Article reporting on same retrospective study already in this review.
2002, Vohra [36]	Article on an RCT that did not appear to eventuate.
2002, Brown [37]	A review of the RCT already in this review
2007, Weeson [38]	No evidence presented on mechanism of raspberry leaf.
2008, Venskuton is et al. [39]	Evidence on different constituents on raspberry leaf when grown in different geographical locations, not on mechanism.
2009, Lans et al. [12]	Use in pets – no evidence of mechanism
2009, Holst et al. [14]	A review of the literature
2009, Holst et al. [40]	Survey of mothers
2010, Tiran [41]	Opinion piece
2012, Hall et al. [42]	Review of CIM for induction. No evidence on mechanism
2011, Trillo et al. [43]	An article on a proposed RCT in Spain that did not appear to proceed
2016, Weed [44]	An opinion piece
2017, Gilmartin [45]	Survey of health care professionals
2019, Munoz Balbontin et al. [46]	A Systemic review of many herbal products used in pregnancy and postnatal review. Only identified 3 studies on raspberry leaf that had already been incorporated in this review.

### Study characteristics

Thirteen studies were included. Five were laboratory studies using animal and human tissue, two were experiments using animals, and six were human studies. Included studies were published between 1941 and 2016. The laboratory studies used rat uteri [29], guinea pig ilium [27], rat and human uteri [25], guinea pig uteri and ilium and frog rectum [24] and cat, dog, rabbit and guinea pig uteri [22]. The two animal studies were RCTs; one using pregnant rats [28] and the other pregnant rats and their offspring [31]. The human studies included a case series [23], three retrospective cohort studies [17, 30, 33], an RCT [16] and one case study [32]. The earliest human study was a case series conducted in 1941 [23] with the remaining published from 1999 with the most recent in 2016. The human studies were conducted in the UK [23], Czech Republic [33], Australia [16, 17], USA [32] and Norway [30]. The findings have been organised under the following headings: laboratory and animal studies and human studies. Table 2 presents a summary of included studies.

**Table 2:** Summary of Results

Year, Authors (in chronological order)	Design	Participants	Methods	Preparation and dosage	Analysis	Effect on Uterus	Effect on other physiology
1941, Burn & Whithell [22]	Laboratory study: in vitro and in vivo	Animal Cat, dog, rabbit and guinea pig uteri (and tissues), spleen, heart and blood vessels	Variety of experiments on both isolated tissue and uteri in situ	Extracts prepared 4 different ways, generally infusion of herb. Author stated dose was 1/100th part of human dose.	unclear	Relaxation effect occasionally followed by contraction	Relaxes intestinal smooth muscle
1941, Whitehouse [23]	Case series	Human 3 postnatal women (Day 5, 7 and 8)	Intrauterine ‘bag’ introduced to uterus to record contractions	Oral dose -	Uterine trace compared (no control)	Relaxation effect, Aid irregular contractions	Very slight fall in systolic pressure
1954, Becket, et al [24]	Laboratory study	Animal Virgin guinea pigs uterine and ilium, Frog rectums	Compared different solutions pharmacological actions in isolated tissue	A variety of preparations, no comparison to human consumption made	unclear	Some constituents were smooth muscle stimulants, others relaxed smooth muscle	Smooth muscle relaxant to ilium and rectum
1970, Bamford, et al [25]	Laboratory study	Animal and human Rat uteri – both pregnant and non-pregnant Human uteri – both pregnant and non-pregnant	Exposed uterine tissue to raspberry leaf and observed for 20 min	Whole leaf used – crushed and infused with saline	unclear	Inhibited contractions of pregnant rats and contracted human uterine tissue in vitro in a coordinated fashion	
1999, Parsons, et al [26]	Retrospective cohort study	Human 108 pregnant women – 57 consumed raspberry leaf, 51 were in control group.	Compared clinical outcomes for mother and infant	Some had tea, some had tablets, some had both, one had tincture. Dose ranged from 1 to 8 cups or tablet per day. 13% commencing 8–28 weeks, 59% from 30 to 34 weeks, 28% from 35 to 39 weeks. Duration over a 1–32 week continuous period.	Descriptive statistics, contingency tables and t-tests	Shorten labour, ↑ term birth, ↓Artificial rupture of membranes, caesareans and instrumental birth No adverse effects.	
2001, Simpson, et al [16]	RCT	Human 192 pregnant women (primips) 96 in treatment group, 96 in placebo group	Compared clinical outcomes for mother and infant, and side effects for cohort of women taking raspberry leaf in pregnancy with those not.	Tablet from 32 weeks gestation for both raspberry leaf and placebo – only difference was addition of 1.2 g extract of raspberry leaf. Conservative dose (as first study of its kind) of 2.4 g / day – considered half dose.	Descriptive & inferential statistical tests depending on variable measured	↓ second stage length, instrumental birth & ARM	
2002, Rojas-Vera, et al [27]	Laboratory study	Animal Guinea pig’s ileum, number not specified	Ileum exposed to raspberry leaf for 5-10 min, washed and re-exposed. Relaxation activity compared 5 min before exposure to after exposure.	Not described	Dose response curve		Smooth muscle relaxant – but dependent on production process of extract of raspberry leaf
2009, Johnson, et al [28]	RCT	Animal Pregnant rats, number not specified	Rats given either raspberry leaf or constituents, then offspring randomly selected checked for onset of puberty	10 mg / kg body weight once breeding confirmed until birth.	Student *t* test and Mann-Whitney rank sum test and Fisher’s exact test	↑gestational length & accelerated reproduction development in offspring	Transgenerational affect
2010, Zheng, et al [29]	Laboratory study	Animal Non-pregnant and pregnant rat uteri (number not specified)	Non-pregnant uteri tissue was exposed to raspberry leave tea, capsules and extract. Based on the results of this only tea was used on pregnant uteri tissue accumulating every 12 min. Synthetic oxytocin was tested similarly.	Authors stated highest concentrations, unlikely to be obtained through consumption by women. However, authors assessment of assumed dosage for humans was suggesting a cup of tea per day (less than common recommendations) and did not stipulate for how long.	Concentration-effect curves	Had more effect on pregnant uteri then non-pregnant uteri. Excited contractions similar to oxytocin in pregnant tissue. Co-treatment of tea and oxytocin enhanced contractility more than oxytocin alone. Variable levels of response from different animals.	
2011, Nordeng, et al [30]	Retrospective cohort study	Human 600 postnatal Norwegian women, 34 took raspberry leaf	Interview using structured questionnaire during postnatal stay in hospital and a review of medical chart.	Not identified	Students *t*-test Pearson’s’ chi-square Linear regression Logistic regression	↑ caesarean risk	
2011, Makaji, et al [31]	RCT	Animal Pregnant rats and their offspring, number not specified	Nulliparous rats received raspberry leaf or variety of derivatives, as soon as breeding confirmed until they birthed. Male and female pups sacrifice from each litter for biopsy of liver	10 mg/kg body weight. Stated this was dose for humans, did not reference the point.	SigmaStat *P < .05*		Female offspring had alterations in liver enzymes
2016, Cheang, et al [32]	Case study	Human Pregnant woman with IDGDM	Hypoglycaemic effects followed use of tea and confirmed by withdrawal and reintroduction of raspberry leaf	Consumed 2 cups of tea at 32 weeks gestation for 3 days.	Naranjo algorithm		↓ Blood glucose levels in a pregnant woman
2016, Bohata & Dostalak [33]	Retrospective cohort study	Human 315 primiparous women, unclear as to how many had raspberry leaf	Women questioned after vaginal birth on methods used to prevent injury	Not specified	Descriptive statistics compared to control group using no preventative birth injury method		No effect on perineal injury

### Laboratory and animal studies

Laboratory studies have focused on the effect of raspberry leaf on smooth muscle including the uterus, ilium and rectum. The earliest laboratory study was in 1941 [22] with three of five studies more than 50 years old [22, 24, 25] and the two more recent studies conducted in 2002 [27] and 2010 [29]. Included studies used a variety of raspberry leaf preparations, dosages, methods of extraction and animal tissues (both in vitro and in vivo) making comparisons difficult.

Laboratory studies which include in vivo and in vitro experiments on a variety of animals, tissues and organs, demonstrate that raspberry leaf contains active constituents that have both relaxation and stimulatory effects on smooth muscle. Rojas-Vera, Patel et al [27] identified at least two active components of raspberry leaf that elicited a relaxant response in guinea pig ileums and Beckett, Belthle et al [24] testing raspberry leaf extracts on a variety of animal tissues identified active components that had smooth muscle stimulant, anticholinesterase, and spasmolytic effects. Burn and Withell [22] identified both stimulatory and relaxant effects on smooth muscle depending on the animal tissue, whether it was in vivo or in vitro and depending on baseline tone of the smooth muscle. More recently Zheng, Pistilli et al [29] examining in vitro, the uteri of nonpregnant and late pregnant rats identified variable effects with raspberry leaf preparation having a more pronounced stimulatory effect on pregnant uterine tissue. This contrasts with the findings of Bamford, Percival et al [25] who applied raspberry leaf extract to the uteri of pregnant and non-pregnant rats and human uterine tissue. In this study the extract had no effect on non-pregnant uterine tissue (rat or human), an inhibitory effect on uterine contractility in pregnant rats and a stimulatory effect on pregnant human uterine tissue.

Rojas-Vera, Patel et al [27] and Beckett, Belthle et al [48] highlight that raspberry leaf extraction methods confound the effects of raspberry leaf. Relaxant effects for Rojas-Vera, Patel et al [27] ranged from none to moderate to a strong dose dependent relaxant effect, using three different elutes. Beckett, Belthle et al [24] found that the component causing spasmolytic effects in their sample antagonised those inspiring smooth muscle stimulation and anticholinesterase effects suggesting that purification methods might be responsible for some of the contradictory effects of raspberry leaf found in some studies [22].

Two randomised controlled trials have been conducted on rats [28, 31] both identifying intergenerational effects. Johnson, Makaji et al [28] found accelerated reproductive development in the female offspring of rats that had been randomly assigned to receive raspberry leaf or specific flavonoids (kaempferol and quercetin). When the offspring were mated, their offspring were more likely to be growth restricted. Their study found that the whole herb had more impact than isolated constituents. Makaji, Ho et al [31] also examined the effects of maternal exposure to raspberry leaf and its constituents, in rats. Their research found that female offspring of rats exposed to either raspberry leaf or some of its constituents experienced long-term alterations in the activity of cytochrome P450 (CYP). In both these animal studies, the rats received raspberry leaf at much higher doses than a woman would normally consume. The interesting similarity of both these studies was that certain intergenerational effects were identified in *female* offspring.

Potential toxicity was identified by Beckett, Belthle et al [24] after active stimulatory components of raspberry leaf were isolated and injected intraperitoneally into mice and chicks in doses equivalent to 0.1 g of raspberry leaf. The agent acted as a central nervous stimulant and cardio vascular toxin causing cyanosis and dilated hearts in mice and convulsions and death in chicks. Burn and Withell [22] found that extracts created with lead acetate (and injected intravenously into cats in doses equivalent to 2 g of raspberry leaf) brought about an initial fall in blood pressure followed by a significant increase. Toxicity was tested by administering extracts to mice orally and intravenously. Lethal doses were achieved with intravenous administration at doses corresponding to 0.4 g of raspberry leaf.

### Human studies

The earliest human study examining the effect of raspberry leaf was published in 1941 [23]. This case series reported on the findings from three women from day five to eight postpartum. Whitehouse [23] measured uterine contractions using an intra-uterine bag after the administration of, “40 grains of crude extract of dried raspberry leaf” in one case, “20 grains” in another and “raspberry leaf tea 20oz. 5%” in the final case (pg 371). In one case the woman was also given pituitrin to stimulate uterine contractions followed by raspberry leaf. In all cases raspberry leaf had a relaxation effect on the uterus with no appreciable impact on blood pressure. While this study makes a contribution to the field, it is lacking in many areas when contemporary appraisal criteria are applied, particulary in relation to reporting of ethical issues including informed consent of the women concerned.

An interesting case study was reported by Cheang, Nguyen et al [32], the most recent study to be published on the topic. In this case study a 38-year-old nulliparous woman with insulin requiring gestational diabetes developed hypoglycaemia after consuming raspberry leaf tea (2 cups per day for three days) at 32 weeks gestation. She reported no change to her diet or physical activity other than the 2 cups of raspberry leaf tea consumed the three days before. The temporal relationship was reinforced by the women’s self-withdrawal and reintroduction of raspberry leaf. The authors used the Naranjo algorithm [49] to test the probability of an adverse drug reaction and concluded that raspberry leaf had probably led to the hypoglycaemic episodes. They recommended that women with gestational diabetes be educated around this and monitor their glucose levels more closely. Case studies such as this have a role to play in describing novel or interesting observations and hypothesising potential relationships but lack scientific rigor and provide no basis for generalising their observation to the wider population.

Three retrospective cohort studies have examined the effect of raspberry leaf on pregnancy outcomes [17, 30, 33]. The study by Bohata and Dostalek [33] was only available as an abstract and thus offered limited information. This study focused on perineal outcomes using several independent variables, of which raspberry leaf was one. It was not clear how many women in the total sample of 315 used raspberry leaf and whether it was ingested or applied externally. There was no statistically significant effect of raspberry leaf on the perineal outcome for participants. A small sub sample (*n* = 34) in a study by Nordeng, Bayne et al [30] focusing more broadly on the use of herbs, reported using raspberry leaf in pregnancy. Women taking raspberry leaf experienced (compared to no use of herbal drugs) a significantly increased rate of caesarean section (23.5% vs 9.1%; adjusted OR 3.47; 95% CI 1.45–8.28). This finding lacks veracity due to the small sample, selection bias, failure to manage potential confounders and lack of detail on dosage, duration, timing and form of raspberry leaf consumed. Parsons, Simpson et al [17] drew on a convenience sample of 108 postnatal women; 57 who reported taking raspberry leaf in pregnancy and 51 who reported they did not. The dose, form, timing and duration of raspberry leaf varied considerably amongst participants who did take raspberry leaf and while this was recorded by the researchers, all those taking any raspberry leaf in pregnancy were analysed as one; the predictor variable being binary (yes or no to taking raspberry leaf in pregnancy). Most women (> 80%) reported a good experience of taking raspberry leaf in pregnancy and would recommend it to a friend. There were no statistically significant differences reported between adverse outcomes including neonatal Apgar score < 6, diastolic blood pressure, meconium stained liquor, transfer to neonatal special or intensive care and postpartum haemorrhage. There were no differences in outcomes for gestation, labour augmentation, epidural, length of first, second and third stages of labour or mode of birth. The null findings of this study may relate to inadequate power with the small sample size. The veracity of these findings is also impacted by selection bias and lack of control for other important potential confounders.

The best available evidence on the effect of raspberry leaf on pregnancy outcomes comes from a double blind, placebo controlled randomised trial by [16]; the only RCT to be conducted on humans in this area. Low risk, nulliparous women (*n* = 192) were randomised to receive two daily doses of raspberry leaf tablets (2 × 1.2 g per day) from 32 weeks gestation or placebo. There were no statistically significant differences between groups on adverse effects including; maternal blood loss, diastolic blood pressure, neonatal birth weight, and meconium stained liquor. *P* values are not reported for tests examining differences in neonatal intensive and special care admissions, occurrence of pregnancy induced hypertension, and side effects/ pregnancy discomforts. No statistically significant differences were found between groups for gestation, augmentation of labour, artificial rupture of membranes, narcotic or epidural analgesia, length of any stage of labour or mode of birth. Potentially *clinically* meaningful differences include a shorter second stage of labour (by almost 10 min) and a smaller proportion of women experiencing forceps birth (19.3% vs. 30.4%) in the raspberry leaf group. The null findings returned for this study may result from the sample size which was powered to detect a substantial 16.6% difference in length of labour or due to the sub-therapeutic dose of raspberry leaf that was used in the study. Women randomised to the treatment arm of this study were given 2.4 g of raspberry leaf per day (in two doses) from 32 weeks gestation which is less than the recommended dose of 4 g daily [11]. While this study offers the strongest evidence available, it has some limitations including lack of detail on the randomisation process (e.g. who prepared the randomised bottles of tablets) and variation in baseline charactersitics of participants (with more participants in the raspeberry leaf group having private maternity care (11.5% vs 5.2%).

## Discussion

Laboratory studies have identified that raspberry leaf contains several active constituents [27], [24] and animal, in vitro and in vivo studies have shown that these have biophysical effects on animal and human tissue, particularly smooth muscle [22, 25] [29]. Raspberry leaf has demonstrated both stimulatory and relaxation effects on smooth muscle depending on a variety of factors including; herbal preparation used [29], method of extraction [27] [24], type of tissue and animal [22], baseline muscle tone [22] and pregnancy status of uterus or uterine tissue [25] [29]. Previous studies have also shown variation in the bioactivity of raspberry leaf by geographical region [39]. Toxic effects in animal studies have only been achieved with intraperitoneal or intravenous injection [22, 24]. While results of animal and in vitro studies must be interpreted with caution because they are not always consistent with human and the in vivo situation, they nonetheless offer valuable information on the efficacy and safety of therapeutics [50].

Raspeberry leaf has potential to interact with other drugs. Makaji, Ho et al [31] found that female offspring of rats exposed to raspberry leaf exhibited alterations in the activity of the enzyme cytochrome (CYP). Investigating six herbs commonly used in pregnancy including raspberry leaf, Langhammer and Nilsen [51] found raspberry leaf (especially ethanolic extract) to be a powerful CYP inhibitor. This has implications for herb-drug interactions with potential to cause unusual sensitivity to drug effects at normal doses [52]. While Cheang, Nguyen et al [32] was the only case study identified in our systematic review that suggested a relationship between raspberry leaf and drug sensitivity (insulin in this case) we should be mindful of the potential for raspberry leaf - drug interactions. Case studies such as this have a role to play in describing novel or interesting observations and hypothesising potential relationships but lack scientific rigor and provide no basis for generalising their observation to the wider population.

Others have warned of the potential for herb-drug interactions including McLay, Izzati et al [53] who conducted a cross-sectional survey with pregnant women (*n* = 889) in Scotland. They found that a high proportion (44.9%) of the women who were taking prescribed medication, were also taking herbal and natural preparations and in these, they identified 34 herb-drug interactions in 12.7% of the women. The herbal and natural products identified in the interactions included aloe, chamomile, cranberry, fish oil, ginger, ginseng, grapefruit, and sage. Raspberry leaf was not implicated though the inhibition of CPY was cited as a potential mechanism in the herb-drug interactions. Authors have also raised concerns that constituents in raspberry leaf (polyphenols) could compete with iron for absorption [54], promoting anaemia in childbearing women taking raspberry leaf. This has not been demonstrated in any studies of raspberry leaf use in pregnancy to date.

The body of evidence informed by human studies on raspberry leaf use in pregnancy does not show any benefit. There is scant evidence from these works to suggest that raspberry leaf has an appreciable effect as a parturient with the only indication coming from the study by Simpson, Parsons et al [16] who identified a clinically (though not statistically) significant difference with women in the raspberry leaf group experiencing a shorter second stage of labour and fewer women experiencing augmentation of labour. Likewise, there is scant evidence to suggest that raspberry leaf has a detrimental effect. While Nordeng, Bayne et al [30] identified an increase in caesarean section amongst the cohort taking raspberry leaf the small sample size, selection bias and lack of control of variables means this result cannot be accepted with any confidence. The case study by Cheang, Nguyen et al [32] highlights the potential for herb-drug interactions and serves as a reminder to clinicians of the importance of taking a thorough medication history from pregnant women which includes the use of herbs and other supplements.

### Limitations of review

Several limitations of this review must be acknowledged. A lack of internationally consistent terminology can impact search strategies in this area. This review focussed on articles published in peer reviewed journals and some have suggested that practitioners and researchers of CIM may not be inclined to publish in these types of journals. Limiting the search to peer review papers however, infers a level of quality. This review also sought only articles published in English, potentially missing relevant research conducted in other countries and in other languages (such as China or India) where the practice of CIM is more mainstream. The decision to limit articles to those published in English was due to the limited resources available to support this study.

There are few contemporary laboratory based studies examining the effects of raspberry leaf on smooth muscle with only two [27], [29] conducted within the last 18 years and only one in the last 10 y [29]. Laboratory procedures and reporting practices have evolved significantly in the last 20 years and there is scope to improve our foundational understanding of the potential effects of raspberry leaf from well conducted laboratory studies which provide detail on the raspberry leaf plant type, dosages, methods of extraction and type of animal tissue and tissue preparation.

This review sought to bring all the relevant empirical research to the table; old and new, in vitro, in vivo, animal and human, which has made synthesis of the findings difficult. Nonetheless this provides a thorough presentation of the state of the art in relation to the biophysical effects, safety and efficacy of raspberry leaf use in pregnancy.

### Implications for future research

This review highlights the need for further research into the effects of raspberry leaf use in human pregnancy. The only randomised controlled study on human subjects in this area used a sub-therapeutic dose of raspberry leaf, taking a conservative approach as it was the first study of this kind. This however, leaves the question of the efficacy of raspberry leaf (at therapeutic levels) un-answered. We suggest that we can have some confidence that raspberry leaf does not have significant detrimental effects given the long history of raspberry leaf use and the large proportion of women that currently use it in pregnancy. There is scope to conduct further research in this area and both clinical trials and well conducted prospective cohort studies would add significantly to the evidence base. Such studies should provide detail on the type, form, dosage, and timing of raspberry leaf consumed and have a sample size that can accommodate sub analyses based on differences in these parameters.

## Conclusion

A large proportion of pregnant women take raspberry leaf in pregnancy with the aim of facilitating an easier birth however, as this review has demonstrated there is a dearth of evidence to inform the practice. In vitro studies have demonstrated biophysical effects on human and animal tissue though these effects are often contradictory. Toxicity has only been demonstrated in animal studies when large amounts of raspberry leaf extract is injected intravenously or intraperitoneally. Human studies have not demonstrated any statistically significant effects. The evidence base is impacted by lack of detail and consistency in preparations, dosage and timing of raspberry leaf (or its constituents) used in the studies.

This integrative review presents the state of the art of the evidence informing the use of raspberry leaf in pregnancy and while we can be reassured by the long history of the practice and lack of documented evidence of harm, contemporary healthcare practice demands that we examine the safety and efficacy of the use of raspberry leaf in pregnancy. Further research is required to provide this information.

## Data Availability

N/A

## Abbreviations

CIM: Complementary and Integrated Medicine
RCT: Randomised controlled trial
NICE: National Institute for Health Care and Excellence
MMAT: Mixed Methods Appraisal Tool
CYP: Cytochrome P450
